# Microcytic hypochromic Anemia is a risk factor for postoperative HAEC: A retrospective study

**DOI:** 10.3389/fsurg.2023.1055128

**Published:** 2023-02-15

**Authors:** Yuanyuan Huang, Hongxia Ren

**Affiliations:** Shanxi Provincial Children's Hospital, Taiyuan, China

**Keywords:** hirschsprung-associated enterocolitis (HAEC), risk factors, microcytic and hypochromic anemias, respiratory infection, preoperative stoma, preoperative history of HAEC, long segment or total colon HSCR

## Abstract

**Background:**

Hirschsprung-associated enterocolitis (HAEC) is a common and life-threatening complication of Hirschsprung's disease (HSCR), which can occur before and after surgery. The aim of this study was to identify the risk factors associated with the development of HAEC.

**Methods:**

We retrospectively reviewed the medical records of HSCR patients admitted to the Children's Hospital of Shanxi Province, China, between January 2011 and August 2021. Diagnosis of HAEC was made using a scoring system with cutoff values ≥4 and included the patient's history, physical examination, and radiological and laboratory findings. The results are shown as frequency (%). The chi-square test was used to analyze a single factor with a significance level of *P *< 0.05. Logistic regression analysis was used to analyze multiple factors.

**Results:**

A total of 324 patients were included in this study, with 266 males and 58 females. In total, 34.3% (111/324) of patients had HAEC, including 85 males and 26 females; 18.9% (61/324) of patients had preoperative HAEC; and 15.4% (50/324) of patients had postoperative HAEC within one year after surgery. Gender, age at definitive therapy, and feeding methods were not found to be associated with preoperative HAEC in univariate analysis. Respiratory infection was associated with preoperative HAEC (*P *= 0.00003). No association was found between gender and age at definitive therapy and postoperative HAEC. Postoperative HAEC was associated with microcytic hypochromic anemia (*P *= 0.00058), preoperative history of HAEC (*P *= 0.00120), the creation of a preoperative stoma (*P *= 0.00097), long segment or total colon HSCR (*P *= 0.00057), and hypoalbuminemia (*P *= 0.03225). Regression analysis showed that microcytic hypochromic anemia (OR=2.716, 95% CI = 1.418–5.203, *P *= 0.003), preoperative history of HAEC (OR=2.814, 95% CI = 1.429–5.542, *P *= 0.003), the creation of a preoperative stoma (OR=2.332, 95% CI = 1.003–5.420, *P *= 0.049), and long segment or total colon HSCR (OR=2.167, 95% CI = 1.054–4.456, *P *= 0.035) were associated with postoperative HAEC.

**Conclusion:**

This study revealed that the incidence of preoperative HAEC at our hospital was associated with respiratory infections. In addition, microcytic hypochromic anemia, preoperative history of HAEC, the creation of a preoperative stoma, and long segment or total colon HSCR were risk factors of postoperative HAEC. The most important finding of this study was that microcytic hypochromic anemia was a risk factor for postoperative HAEC, which has been rarely reported. Further studies with larger sample sizes are necessary to confirm these findings.

## Introduction

Hirschsprung's disease (HSCR) is a complex disorder of the enteric nervous system (ENS) characterized by defective migration, proliferation, differentiation, and survival of neural crest cells (NCC), resulting in the complete absence of ganglion cells in the intestinal tract (1). Mortality of HSCR is significantly reduced after surgical removal of the aganglionic segments. However, postoperative complications such as intestinal dysfunction remain a major problem in pediatric patients. Almost 42% of patients suffered a decline in their quality of life following the operation (2, 3). Hirschsprung-associated enterocolitis (HAEC) is a life-threatening complication of HSCR with high morbidity and mortality. Several risk factors of preoperative and postoperative HAEC have been reported, but there is no consensus. In this study, we further investigated the risk factors of HAEC.

## Materials and methods

A systematic search of the case retrieval system of the Children's Hospital of Shanxi Province, China, was performed for cases between January 2011 and August 2021 to identify relevant patients. The data collected included gender, age at definitive therapy, methods for feeding, respiratory infection, microcytic hypochromic anemia, preoperative history of HAEC, creation of a preoperative stoma, long segment or total colon HSCR, and hypoalbuminemia. All HSCR patients received rectal irrigation before the surgery. The surgical method used at our hospital was Soave's procedure including transanal or laparoscopic-assisted Soave's procedure, and children with preoperative stoma underwent laparotomy to close the functional stoma.

The inclusion criteria for this study were as follows: ① the children had clinical manifestations such as abdominal distention, chronic constipation, or neonatal obstruction; ② the primary diagnosis of HSCR was confirmed by digital rectal examination, contrast enema, and anorectal manometry; ③ pathological examination before surgery or by intraoperative frozen-section examination was used to establish definite diagnosis, and all patients underwent pathological biopsy after surgery in accordance with the diagnosis of HSCR. The primary diagnosis of HSCR at our hospital was based on clinical symptoms and auxiliary examination ①+②. The diagnosis of all the patients included in this study was based on 2462;. The exclusion criteria of this study were as follows: ① no surgical treatment was provided; ② children with HSCR who were lost to follow-up within one year after pull-through surgery.

This study was approved by the hospital ethics committee of the Children's Hospital of Shanxi Province, China.

### HAEC

HAEC was determined using the Delphi scoring system with cutoff values ≥4, and included the patient's history (diarrhea with explosive stool, diarrhea with foul-smelling stool, diarrhea with bloody stool, and history of enterocolitis), physical examination (explosive discharge of gas and stool on rectal examination,distended abdomen, decreased peripheral perfusion, lethargy and fever), radiographic findings/upright plain abdominal film (multiple air-fluid levels, dilated loops of bowel, sawtooth appearance with irregular mucosal lining, cutoff sign in rectosigmoid with the absence of distal air, pneumatosis), and laboratory findings (leucocytosis, cell nuclei shifted to left). The period of follow-up was one year after the operation.

## Statistical analysis

IBM SPSS Statistics version 26.0 was used for statistical analysis. Data are presented as numbers and percentages for categorical variables. The chi-square test was used to evaluate the difference in risk factors between the groups. The variables with significant differences in the univariate analysis were included in the multivariate logistic regression analysis. A *P* < 0.05 was considered statistically significant.

## Results

### Baseline characteristics

A total of 324 HSCR patients were included in this study, with 266 males and 58 females. Among these patients, 111 had HAEC including 61 with preoperative HAEC and 50 with postoperative HAEC. The age at radical resection was usually > 3 months (69.8%), and the patients were mainly on non-exclusive breastfeeding (63.9%). The respiratory status of the child was well managed during the perioperative period (85.8%). The majority of postoperative HAEC patients had no microcytic hypochromic anemia (61.7%), preoperative history of HAEC (74.4%), and preoperative stoma (88.0%), and showed short-segment aganglionosis (79.9%) and hypoalbuminemia (69.1%) (Table 1).

**Table 1:** Baseline characteristics of HAEC in children with HSCR.

Characteristics	*n* (%)
**Gender**	
Male	266 (82.1)
Female	58 (17.9)
**Age at definitive therapy**	
≤3 months	98 (30.2)
> 3 months	226 (69.8)
**Respiratory infection**	
Yes	46 (14.2)
No	278 (85.8)
**Feeding methods**	
Exclusive breastfeeding	117 (36.1)
Non-exclusive breastfeeding	207 (63.9)
**Microcytic hypochromic anemia**	
Yes	124 (38.3)
No	200 (61.7)
**Preoperative history of HAEC**	
Yes	83 (25.6)
No	241 (74.4)
**Preoperative stoma**	
Yes	39 (12.0)
No	285 (88.0)
**Long segment or total colon HSCR**	
Yes	65 (20.1)
No	259 (79.9)
**Hypoalbuminemia**	
Yes	224 (69.1)
No	100 (30.9)

### Univariate analysis of preoperative HAEC

Overall, 18.8% of the HSCR patients had preoperative HAEC. Gender, age at definitive therapy, and feeding methods were not associated with preoperative HAEC. Respiratory infection was associated with preoperative HAEC (*P *= 0.00003) (Table 2).

**Table 2:** Analysis of preoperative HAEC in children with HSCR.

	HAEC	Non-HAEC	*χ* ^2^	*P* value
	(*n*, %)	(*n*, %)		
**Gender**				
Male	45 (73.8)	221 (84.0)	3.868	0.049
Female	16 (26.2)	42 (16.0)		
**Age at definitive therapy**				
≤3 months	16 (26.2)	82 (31.2)	0.575	0.448
>3 months	45 (73.8)	181 (68.8)		
**Respiratory infection**				
Yes	19 (31.1)	27 (10.3)	17.723	0.00003
No	42 (68.9)	236 (89.7)		
**Feeding methods**				
Exclusive breastfeeding	20 (32.8)	97 (36.9)	0.360	0.549
Non-exclusive breastfeeding	41 (67.2)	166 (63.1)		

### Univariate analysis of postoperative HAEC

Additionally, 15.4% of the HSCR patients had postoperative HAEC. No association was found between gender, age at definitive therapy, and postoperative HAEC. Postoperative HAEC was associated with microcytic hypochromic anemia (*P *= 0.00058), preoperative history of HAEC (*P *= 0.00120), the creation of a preoperative stoma (*P *= 0.00097), long segment or total colon HSCR (*P *= 0.00057), and hypoalbuminemia (*P *= 0.03225) (Table 3).

**Table 3:** Analysis of postoperative HAEC in children with HSCR.

	HAEC (*n*, %)	Non-HAEC (*n*, %)	χ^2^	*P* value
**Gender**				
Male	40 (80.0)	226 (82.5)	0.177	0.674
Female	10 (20.0)	48 (17.5)		
**Age at definitive therapy**				
≤3 months	14 (28.0)	84 (30.7)	0.141	0.707
>3 months	36 (72.0)	190 (69.3)		
**Microcytic hypochromic anemia**				
Yes	30 (60.0)	94 (34.3)	11.816	0.00059
No	20 (40.0)	180 (65.7)		
**Preoperative history of HAEC**				
Yes	22 (44.0)	61 (22.3)	10.485	0.001
No	28 (56.0)	213 (77.7)		
**Preoperative stoma**				
Yes	13 (26.0)	26 (9.5)	10.887	0.00097
No	37 (74.0)	248 (90.5)		
**Long segment or total colon HSCR**				
Yes	19 (38.0)	46 (16.8)	11.863	0.00057
No	31 (62.0)	228 (83.2)		
**Hypoalbuminemia**				
Yes	41 (82.0)	183 (66.8)	4.585	0.032
No	9 (18.0)	91 (33.2)		

### Multivariate analysis of postoperative HAEC

The variables with statistically significant differences in the univariate analysis of postoperative HAEC were included in the binary logistic regression analysis. Microcytic hypochromic anemia (OR=2.716, 95% CI = 1.418–5.203, *P *= 0.003), preoperative history of HAEC (OR=2.814, 95% CI = 1.429–5.542, *P *= 0.003), the creation of a preoperative stoma (OR=2.332, 95% CI = 1.003–5.420, *P *= 0.049), and long segment or total colon HSCR (OR=2.167, 95% CI = 1.054–4.456, *P *= 0.035) were the risk factors of postoperative HAEC (Table 4).

**Table 4:** Logistic multivariate analysis of postoperative HAEC in children with HSCR.

Factor	B	S.E.	Wald	*P* value	*OR*	95% *CI*
Microcytic hypochromic anemia	0.999	0.332	9.075	0.00259	2.716	1.418-5.203
Preoperative history of HAEC	1.035	0.346	8.951	0.00277	2.814	1.429-5.542
Preoperative stoma	0.847	0.430	3.873	0.049	2.332	1.003-5.420
Long segment or total colon HSCR	0.773	0.368	4.422	0.035	2.167	1.054-4.456
Hypoalbuminemia	0.373	0.421	0.786	0.375	1.452	0.637-3.310

## Discussion

HSCR is one of the most common and severe diseases threatening neonatal growth and development. Over the past several decades, radical pull-through surgery has revolutionized the survival of children with HSCR. However, HAEC is one of the most common and serious complications of HSCR that affects the quality of life of young children. Previous reports revealed that HAEC occurred in 6%–50% of the patients preoperatively and in 2%–35% postoperatively. The incidence of preoperative HAEC at our hospital was 18.8%, and postoperative HAEC was 15.4%, which is similar to previous reports. The etiology and mechanism of HAEC are not fully understood, and most of the studies have focused on gut microbiota, intestinal epithelial barrier function, and genes (4–7). It is essential to identify the risk factors of HAEC and provide prompt clinical interventions.

This study revealed that gender and age at definitive therapy were not associated with preoperative and postoperative HAEC. During the management of HAEC, we found that prompt diagnosis and intervention were more important than the choice of age at definitive therapy. For example, early diagnosis and rectal irrigation were critical to resolve fecal stasis and significantly improve the intestinal environment in HSCR. The inflammatory state of the intestine can be effectively alleviated by metronidazole and broad-spectrum antibiotics (8). A recent study suggested that exclusive breastfeeding is a novel protective factor for preoperative and postoperative HAEC (9), but we did not find any association between feeding methods and preoperative HAEC. Hence, further studies are needed to clarify the role of breastfeeding in HAEC. Respiratory infection is a common disease in pediatrics during the perioperative period. Affected children often have clinical manifestations such as cough, sputum production, and fever, together with or without increased Leukocytes (>10.0  ×  10^9^/l), Neutrophils (>8.00  ×  10^9^/l), and C-reactive protein (>10 mg/l). Respiratory tract infection in children during the perioperative period was reported as a risk factor for preoperative HAEC, probably because respiratory tract infections can increase interferon (IFN)-*γ* and trigger intestinal homeostasis, which in turn leads to the occurrence of HAEC (10). Our study also supports this finding, and neutrophils may play a role in the development of HAEC (11).

A significant relationship between microcytic hypochromic anemia and postoperative HAEC was observed in this study, which has been rarely reported before (OR=2.716, *P *= 0.003). It is believed that dysregulation of gut microbiota could be a potential mechanism for HAEC, and previous studies also showed lower hematocrit, along with increased Proteobacteria and decreased Firmicutes (12). This finding offers a potential microbiological explanation for this condition. It also suggests that the impaired gastrointestinal function in HAEC could lead to bleeding and iron malabsorption resulting in anemia, which may be an independent risk factor for intestinal inflammation and damage. A study conducted on a neonatal murine model revealed that anemia may contribute to gut inflammation and barrier disruption by altering macrophage function. Moreover, anemia reduces the ability of red blood cells to carry oxygen and nutrients, resulting in severe hypoxia in the intestinal mucosa, which may lead to abnormal intestinal homeostasis and immune regulation changes (13). This could be the underlying mechanism for the pathogenesis of various intestinal inflammatory diseases. At our center, we actively give iron supplement treatment for 1–2 months; if the anemia is severe (hemoglobin <90 g/L, neonatus:hemoglobin <120 g/L), we also give blood transfusions to the child. After these treatments, anemia will have a varying degree of improvement, and when the condition is corrected (hemoglobin >90 g/L, neonatus: hemoglobin >120 g/L), we perform radical surgery or discharge the children from the hospital. However, the mechanism of microcytic hypochromic anemia and postoperative HAEC remains unclear, and this conclusion was previously reported in only one case. Hence, opportunities exist to provide a promising new treatment strategy.

A preoperative history of HAEC (OR=2.814, 95% CI = 1.429–5.542, *P *= 0.003) was found to increase the likelihood of developing postoperative HAEC. This is consistent with the findings of previous studies, which also showed that preoperative history of HAEC is a risk factor for postoperative HAEC (14). Another study was conducted to compare the fecal bacterial and fungal communities of children who developed HAEC with HSCR patients who never had enterocolitis. The results of the study revealed that the bacterial composition in the HAEC group showed a modest decrease in Firmicutes and Verrucomicrobia, while the numbers of Bacteroides and Proteus increased. In addition, the diversity of fecal fungi was significantly decreased in the HAEC group, and the number of Candida sp. was increased, while the numbers of Malassezia and Saccharomyces sp. were reduced. These findings suggested that dysbiosis of the gut microbial ecosystem in HAEC patients makes patients with a preoperative history of HAEC potentially more susceptible to HAEC later (15). Three children at our hospital had three or more HAEC occurrences within one year after pull-through surgery, all of them had a preoperative history of HAEC, and a lack of early effective intervention. Although we performed effective surgical treatment later, recurrent HAEC slowed the growth and development of these children and decreased their quality of life. Thus, effective early identification and clinical intervention may help reduce the occurrence and progression of enterocolitis.

The creation of a preoperative stoma (OR=2.332, 95% CI = 1.003–5.420, *P *= 0.049) was associated with postoperative HAEC. The reason pediatric surgeons usually create a preoperative functioning stoma is that the children have extremely poor intestinal function before surgery, which is usually caused by the disorders of intestinal microbiota, mucus barrier, and immune system, and this pro-inflammatory intestinal state often requires repeated medical interventions (14). Therefore, patients need the creation of a stoma before a definitive pull-through operation for HSCR in order to provide sufficient rest and relief to the intestine. However, the primary mechanism between them remains unknown.

Several studies reported that segment length is associated with pre- and postoperative HAEC (16). In this study, we found that long segment or total colon HSCR (OR=2.167, 95% CI = 1.054–4.456, *P *= 0.035) is a risk factor of postoperative HAEC, which has been previously reported (17). The reason is mainly the absence of ganglion cells and hypertrophy of non-myelinated peripheral nerve fibers involving a wider range of colon segments. The longer the lesion colon segment, the greater impact on the overall peristaltic function of the colon, and the greater the risk of fecal stasis. This is the main reason for developing HAEC. Treatment of the functional obstruction may effectively relieve or prevent HAEC, such as laxatives or enemas and intrasphincteric botulinum toxin injections for postoperative defecation.

We hypothesized that hypoalbuminemia is one of the risk factors for postoperative HAEC. The univariate results of this study suggested that combined hypoalbuminemia was associated with the occurrence of postoperative HAEC, but the logistic multivariate analysis did not show statistical significance (*P *= 0.375 > 0.05). Previous studies have also supported this conclusion (18). Another study revealed that preoperative albumin levels may be the best predictor of mortality after colorectal cancer surgery (19). However, whether this index is related to the radical recovery after HSCR needs further study.

## Conclusion

This study showed that respiratory infection was associated with preoperative HAEC at our hospital. Microcytic hypochromic anemia, preoperative history of HAEC, the creation of a preoperative stoma, and long segment or total colon HSCR were risk factors for postoperative HAEC. The most important finding of this study is that microcytic hypochromic anemia is a risk factor for postoperative HAEC, which needs the attention of pediatric surgeons worldwide. However, its potential mechanism and causes need further research with large samples and in multiple centers.

## Data Availability

The raw data supporting the conclusions of this article will be made available by the authors, without undue reservation.

## References

[1] Klein M Varga I. Hirschsprung's disease-recent understanding of embryonic aspects, etiopathogenesis and future treatment avenues. Medicina (Kaunas). (2020) 56(11):611. 10.3390/medicina56110611 33202966 PMC7697404

[2] Pan W Goldstein AM Hotta R. Opportunities for novel diagnostic and cell-based therapies for hirschsprung disease. J Pediatr Surg. (2022) 57(9):61–8. 10.1016/j.jpedsurg.2021.10.049 34852916 PMC9068833

[3] Davidson JR Kyrklund K Eaton S Pakarinen MP Thompson DS Cross K Long-term surgical and patient-reported outcomes of hirschsprung disease. J Pediatr Surg. (2021) 56(9):1502–11. 10.1016/j.jpedsurg.2021.01.043 33706942

[4] Dariel A Grynberg L Auger M Lefèvre C Durand T Aubert P Analysis of enteric nervous system and intestinal epithelial barrier to predict complications in Hirschsprung's Disease. Sci Rep. (2020) 10(1):21725. 10.1038/s41598-020-78340-z 33303794 PMC7729910

[5] Chantakhow S Khorana J Tepmalai K Boonchooduang N Chattipakorn N Chattipakorn SC. Alterations of gut Bacteria in hirschsprung disease and hirschsprung-associated enterocolitis. Microorganisms. (2021) 9(11):2241. 10.3390/microorganisms9112241 34835367 PMC8623574

[6] Bachetti T Rosamilia F Bartolucci M Santamaria G Mosconi M Sartori S The OSMR gene is involved in hirschsprung associated enterocolitis susceptibility through an altered downstream signaling. Int J Mol Sci. (2021) 22(8):3831. 10.3390/ijms22083831 33917126 PMC8067804

[7] Zhang H Zhao JL Zheng Y Xie XL Huang LH Li L Correlation analysis of IL-11 polymorphisms and hirschsprung disease subtype susceptibility in southern Chinese children. BMC Med Genomics. (2021) 14(1):21. 10.1186/s12920-020-00867-x 33468134 PMC7814452

[8] Gosain A Frykman PK Cowles RA Horton J Levitt M Rothstein DH American Pediatric surgical association hirschsprung disease interest group. Guidelines for the diagnosis and management of hirschsprung-associated enterocolitis. Pediatr Surg Int. (2017) 33(5):517–21. 10.1007/s00383-017-4065-8 28154902 PMC5395325

[9] Tang W Su Y Yuan C Zhang Y Zhou L Peng L Prospective study reveals a microbiome signature that predicts the occurrence of post-operative enterocolitis in hirschsprung disease (HSCR) patients. Gut Microbes. (2020) 11(4):842–54. 10.1080/19490976.2020.1711685 31944159 PMC7524399

[10] Li S Tu JR Zhang L Luo ZB Yang DH Li K Correlation between upper respiratory tract infection and Hirschsprung's Disease associated enterocolitis. Chin J Exp Surg. (2021) 38(02):343–6. 10.3760/cma.j.cn421213-20200720-01253

[11] Drury B Hardisty G Gray RD Ho GT. Neutrophil extracellular traps in inflammatory bowel disease: pathogenic mechanisms and clinical translation. Cell Mol Gastroenterol Hepatol. (2021) 12(1):321–33. 10.1016/j.jcmgh.2021.03.002 33689803 PMC8166923

[12] Ho TTB Kumar A Louis-Jacques AF Dishaw LJ Yee AL Groer MW. The development of intestinal dysbiosis in anemic preterm infants. J Perinatol. (2020) 40(7):1066–74. 10.1038/s41372-020-0599-z 31992818 PMC7319903

[13] Arthur CM Nalbant D Feldman HA Saeedi BJ Matthews J Robinson BS Anemia induces gut inflammation and injury in an animal model of preterm infants. Transfusion. (2019) 59(4):1233–45. 10.1111/trf.15254 30897226 PMC6525338

[14] Pruitt LCC Skarda DE Rollins MD Bucher BT. Hirschsprung-associated enterocolitis in children treated at US children's Hospitals. J Pediatr Surg. (2020) 55(3):535–40. 10.1016/j.jpedsurg.2019.10.060 31836243 PMC7780549

[15] Frykman PK Nordenskjöld A Kawaguchi A Hui TT Granström AL Cheng Z Characterization of bacterial and fungal microbiome in children with hirschsprung disease with and without a history of enterocolitis: a multicenter study. PLoS One. (2015) 10(4):e0124172. 10.1371/journal.pone.0124172 25909773 PMC4409062

[16] Romo Muñoz MI Martínez de Aragón A Núñez Cerezo V Udaondo C Sellers M Barrena S Risk factors associated with the development of enterocolitis in Hirschsprung's Disease. Cir Pediatr. (2018) 31(1):34–8. 29419957

[17] Chung PHY Yu MON Wong KKY Tam PKH. Risk factors for the development of post-operative enterocolitis in short segment Hirschsprung's Disease. Pediatr Surg Int. (2019) 35(2):187–91. 10.1007/s00383-018-4393-3 30386902

[18] Yulianda D Sati AI Makhmudi A. Risk factors of preoperative hirschsprung-associated enterocolitis. BMC Proc. (2019) 13(Suppl 11):18. 10.1186/s12919-019-0172-y 31890011 PMC6912936

[19] Truong A Hanna MH Moghadamyeghaneh Z Stamos MJ. Implications of preoperative hypoalbuminemia in colorectal surgery. World J Gastrointest Surg. (2016) 8(5):353–62. 10.4240/wjgs.v8.i5.353 27231513 PMC4872063

